# Characterization of Carbon-Contaminated B_4_C-Coated Optics after Chemically Selective Cleaning with Low-Pressure RF Plasma

**DOI:** 10.1038/s41598-018-19273-6

**Published:** 2018-01-22

**Authors:** H. Moreno Fernández, D. Rogler, G. Sauthier, M. Thomasset, R. Dietsch, V. Carlino, E. Pellegrin

**Affiliations:** 1 CELLS-ALBA, Carrer de la Llum 2-26, E-08290 Cerdanyola del Valles, Spain; 2 AXO DRESDEN GmbH, Gasanstaltstr. 8B, D-01237 Dresden, Germany; 3 ICN2, UAB Campus, E-08193 Bellaterra, Spain; 4 SOLEIL Synchrotron, Saint Aubin BP48, L’Orme des Merisiers, Gif-sur-Yvette Cedex, F-91192 France; 5 ibss Group Inc., 111 Anza Bld., Suite 110, Burlingame, CA 94010 USA

## Abstract

Boron carbide (B_4_C) is one of the few materials that is expected to be most resilient with respect to the extremely high brilliance of the photon beam generated by free electron lasers (FELs) and is thus of considerable interest for optical applications in this field. However, as in the case of many other optics operated at light source facilities, B_4_C-coated optics are subject to ubiquitous carbon contaminations. Carbon contaminations represent a serious issue for the operation of FEL beamlines due to severe reduction of photon flux, beam coherence, creation of destructive interference, and scattering losses. A variety of B_4_C cleaning technologies were developed at different laboratories with varying success. We present a study regarding the low-pressure RF plasma cleaning of carbon contaminated B_4_C test samples via inductively coupled O_2_/Ar, H_2_/Ar, and pure O_2_ RF plasma produced following previous studies using the same ibss GV10x downstream plasma source. Results regarding the chemistry, morphology as well as other aspects of the B_4_C optical coating before and after the plasma cleaning are reported. We conclude that among the above plasma processes only plasma based on pure O_2_ feedstock gas exhibits the required chemical selectivity for maintaining the integrity of the B_4_C optical coatings.

## Introduction

Boron carbide (B_4_C) as an engineering material has a long track record of applications in various fields of applications^1^, although some of its polytypism is still not under full control. Nevertheless, optical engineering is presently increasing the usage of B_4_C as an optical coating material for a large range of optical application^2^, including beamline optics in accelerator-based light sources such as synchrotron as well as free electron laser (FEL) facilities together with materials of similar hardness such as, e.g., SiC, cubic BN etc. where B_4_C is preferably used in the soft x-ray photon energy range (0.5 to 2.5 keV photon energy) while SiC is employed in the hard x-ray range (2 to 20 keV). This usage includes single coatings as well as multilayer mirror coatings based on, e.g., SiC/B_4_C multilayers.

While the cleaning of carbon contaminations of beamline optics based on metallic reflective coatings has become a widespread activity^3–5^, more complex optical surfaces and coatings such as, e.g., amorphous carbon, diamond-like carbon (DLC), silicon carbide (SiC), and boron carbide (B_4_C) are now at the center of interest due to their unique capability of withstanding the high brilliance of the pulsed photon beam emitted from FEL light sources, which - at the same time - leads to increased carbon contamination rates on optical surfaces.

On the other hand, the extreme requirements imposed by FEL applications on the quality of beamline optical components and their preservation (especially when it comes to coherence-based experiments) imperatively call for the development of *in-situ* cleaning processes that warrant for the safe, efficient, and well-understood cleaning of FEL optical components with carbon-based optical coatings (such as, e.g., the above B_4_C, SiC etc.). Obviously, as carbon is present in both the coating as well as the surface contamination the cleaning technique to be used should necessarily include an inherent chemical selectivity for distinguishing these two different carbon species from each other in order to prevent any damage of the optical coating. To our present knowledge, several attempts have been performed so far regarding a cleaning of B_4_C-coated optics using oxygen- as well as ozone-based *in-situ* and *ex-situ* techniques, but all of them so far did lead to a degraded B_4_C coating and were thus not deemed acceptable^6^.

In this paper, we describe an experimental approach based on the low-pressure RF downstream plasma cleaning of various B_4_C-coated test objects, which includes a variation of the plasma chemistry by a variation of the plasma feedstock gases. Although the employed experimental procedure is similar to the one described in previous studies^7,8^, the present results turn out to be completely different from those based on the cleaning of noble and non-noble metallic reflective optical coatings. In more detail, the results obtained give evidence for a satisfactory cleaning performance only for pure O_2_ plasma, while an O_2_/Ar feedstock gas mixture results into an efficient carbon cleaning as well, but combined with significant degradation of the B_4_C layer. The latter effect is even more pronounced in the case of Ar/H_2_ feedstock gas mixtures, leading to a substantial reduction of the B_4_C layer thickness. We tentatively attribute these findings to a detrimental effect from the *kinetic* contribution from the plasma induced by especially the Ar species within the plasma. Thus, avoiding plasma species with elevated masses (such as, e.g., Ar) reduces the *kinetic* contribution within the plasma cleaning process, thus emphasizing the *chemical* contribution to the cleaning process while at the same time reducing its detrimental effect on the optical coating. In addition, we did find evidence for the plasma-induced surface conversion process of the B_2_O_3_ phase inherent to the B_4_C bulk layer into surface boron oxy-carbides, resulting into a significant reduction of cleaning rates.

## Experiment

### Coating processes

#### B_4_C coating

Amorphous B_4_C-layers with a nominal thickness of 30 ± 5 nm were fabricated by the AXO Dresden GmbH company (Dresden, Germany) on standard Si(100) wafers (10 × 10 mm^2^ size) and optically polished single-crystalline Si substrates (<0.2 nm rms micro-roughness). All substrates were provided with a 2 nm (nominal thickness) Cr buffer layer between the Si substrate and the B_4_C coating. The B_4_C layer was deposited using a Dual Ion Beam Sputtering (DIBS) device from Roth & Rau AG (IONSYS1600).

#### Amorphous carbon contamination coating

For the deposition of amorphous carbon (a-C) layers – i.e., for simulating a photon-beam induced carbon contamination - onto the above B_4_C test samples a commercial e-beam deposition chamber has been used, that allows for a deposition of about 180 nm of carbon within about 200 seconds deposition time. Such an a-C thickness usually provides a sufficiently long cleaning time for the analysis and optimization of the plasma cleaning process.

### Si wafer and mirror test objects and systematic approach

#### Si test wafers

Si(100) wafer pieces, coated with an amorphous B_4_C layer (see Fig. S1; Supplementary Information file) were used in order to characterize changes in the B_4_C surface chemistry as induced by the plasma treatment using x-ray photoemission spectroscopy (XPS). In addition, an energy-dispersive x-ray analysis (EDX) and/or scanning electron microscopy (SEM) analysis was performed on these Si test wafers in order to obtain information on the bulk versus surface chemical stoichiometry as well as changes in the surface morphology of the B_4_C coatings. A partial/central amorphous carbon coating spot was applied in order to investigate the influence of the direct exposure of the plasma onto the B_4_C coating (i.e., with or without the amorphous carbon top coating).

#### Optical Test mirrors

The second type of test objects consists of single-crystalline Si substrates with one inch diameter and an optical polishing with a surface micro-roughness of less than 0.2 nm rms. As in the case of the above Si(100) test wafers, the same B_4_C coating has been applied (see Fig. S2). Also here, a partial amorphous carbon coating has been applied in order to investigate the influence of the direct exposure of the plasma onto the B_4_C coating. The surface micro-roughness measurements on these test mirrors were performed using a standard ZYGO interference microscope setup.

As in the previous studies^7–9^ a standard quartz crystal microbalance (QCM) including gold-coated and a-C contaminated quartz crystals was used to determine the carbon removal rate during the plasma process for optimizing the plasma operation parameters towards the required optimum carbon cleaning rates.

### Cleaning test setup

The test chamber setup for the plasma cleaning consists of a custom UHV chamber with a base pressure of 3 × 10^−7^ mbar (unbaked). The complete cleaning chamber setup including the plasma source and plasma diagnostics are described in detail in a previous study^7^.

### GV10x inductively coupled RF plasma source

A commercial RF gun (model GV10x Downstream Asher, made by ibss Group, Inc., Burlingame, CA 940101, USA) has been used, based on the inductive coupling of the RF into the plasma tube inside the plasma source.

The plasma parameters used for the different cleaning runs are given in Table 1. Typical cleaning times required for an O_2_/Ar or pure O_2_ plasma were in the range of 2.5 hours, whereas the corresponding cleaning times for an Ar/H_2_ plasma were in the range of 12.5 hours, starting from a-C coatings with the same thickness. This significant difference in terms of carbon cleaning rate between oxygen- and hydrogen-based downstream plasma by a factor of up to roughly seven could already be observed in previous studies^7–9^.

**Table 1:** Plasma parameters used for the different cleaning runs.

Gas Mixture	Working Pressure [mbar]	Feedstock Gas Ratio	RF Power [W]	Optical Emission Lines from Radicals	Emission Line Ratio
O_2_/Ar	0.005	Ar 10% O_2_ 90%	100	Ar I 750.4 nm	O* 95% Ar 5%
H_2_/Ar	0.002	H_2_ 65% Ar 35%	100	H I (H*) 656.3 nm	Ar 93% H* 7%
O_2_	0.006	O_2_ 100%	100	O I (O*) 777.2 nm	—

### B_4_C bulk thin film and surface characterization

The B_4_C test samples were analyzed by X-Ray Reflectometry (XRR) using Cu Kα radiation (E_Ph_ = 8.04132 eV photon energy) before and after amorphous carbon coating plus subsequent RF plasma cleaning in order to detect changes in the B_4_C layer thickness and in the roughness of the B_4_C/air interface. Simulations of the XRR results were performed using the IMD program^10^. Changes regarding the B_4_C sample surface chemistry were analyzed by XPS using a SPECS Phoibos 150 electron energy analyzer in conjunction with a monochromatized Al Kα x-ray source.

## Results and Discussion

### O_2_/Ar plasma cleaning

In Fig. S3, we show B_4_C-coated Si test wafers after the a-C deposition (180 nm thickness) as well after the subsequent cleaning with an O_2_/Ar plasma. As can be seen from the visual appearance of the plasma-processed test wafer, a residue from the a-C spot can still be observed, that could not be fully removed without a visual degradation of the blank B_4_C coating. This is in stark contrast to previous findings on metallic optical coatings, where a complete cleaning of the metal surface could be performed (i.e., without any visual residues)^7–9^ and indicates the formation of a chemically stable interface layer that appears to be more persistent with respect to the oxidative cleaning by the O_2_/Ar plasma.

#### X-ray Photoelectron Spectroscopy (XPS) analysis from B_4_C-coated Si test wafer

**Figure 1:**
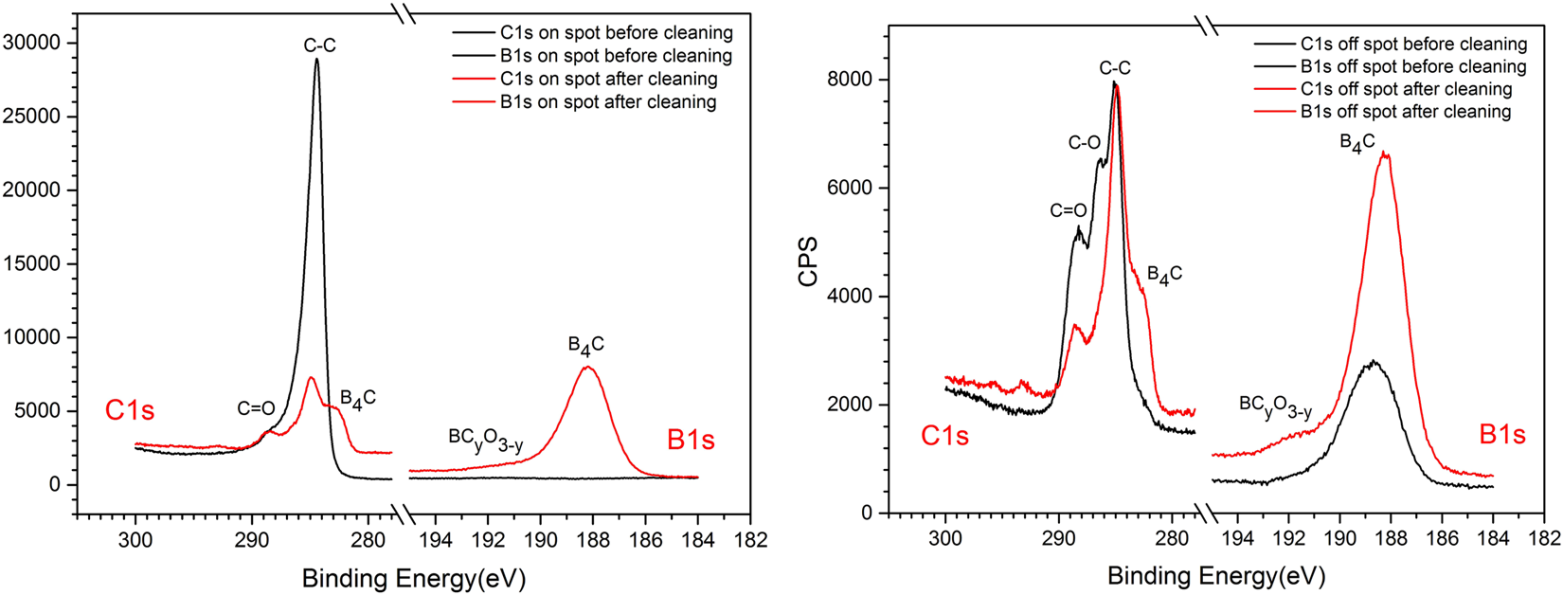
High-resolution C1s and B1s XPS spectra of the B_4_C-coated test wafer before (black solid lines) and after O_2_/Ar RF plasma cleaning (red solid lines). Left panel: XPS data taken on the amorphous carbon contamination spot. Right panel: XPS data taken off the amorphous carbon contamination spot on the bare B_4_C coating.

Figure 1 shows the XPS high resolution spectra from the Si test wafers shown in Fig. S3, with the measurements being performed off as well as on the a-C spot before and after the O_2_/Ar plasma cleaning. We first consider the “on spot” spectra before/after the plasma cleaning. Here, one can clearly distinguish the absence and the appearance of the B1s XPS line before and after the plasma processing, respectively, together with a significant reduction of the C1s core level line.

The cleaning efficiency becomes clear from the increase (reduction) of the B1s (C1s) lines for the “on spot” via the cleaning process, as well as from the overall similarity of the B1s and C1s lines between the “on spot” and “off spot” locations after cleaning. Taking a closer look at the spectra, one can assign the different constituents of the XPS lines as shown in Table 2. These assignments are in good agreement with the previous XPS study by Jacobsohn *et al*. and Jacques *et al*.^11,12^.

**Table 2:** XPS core level line assignments (“BOC” refers to boron oxy-carbides).

XPS line	C1s				B1s				O1s
B.E. [eV]	282.58	284.94	286.6	288.58	188.12	189.49	191.40	193.3	533.0
Assignment	C in B_4_C	C-C	C-O (e.g., in BOC)	C=O	B-B and B-C in B_4_C	B in BC_2_O	B in BCO_2_	B in B_2_O_3_	O in B_2_O_3_

According to the XPS line assignments given in Table 2, one can interpret the high resolution spectra in such a way that the O_2_/Ar plasma surface treatment leads to:

- A substantial reduction of the C-C C1s peak (“on spot” and “off spot”) as well as the C-O and the C=O peaks (“off spot”), that are all related to adventitious or purposeful surface artifacts.
- The appearance of the B-B and B-C B1s line and the associated B-C peak in the C1s spectra (“off spot” and “on spot”) being due to B and C in bulk B_4_C, respectively.
- A weak B1s peak at 191.4 eV B.E. due to the occurrence of boron oxy-carbide (BOC).

On the other hand, we observe for the post-treatment spectra that the spectral fingerprint of especially the C1s XPS lines *invariably* exhibits the three peaks associated with B_4_C, C-C, and C=O (with some minor shoulder at 286.6 eV due to C-O). This indicates the formation of a chemically stable interface layer at the B_4_C surface that is either resilient with respect to the plasma surface interaction and/or is a direct result from the latter. Similar results from different plasma treatments will be shown in the next sections.

Using the same samples and systematics as used for the high resolution XPS spectra in Fig. 1, Fig. S7 shows the XPS survey spectra for the “on spot” and “off spot” sample locations before and after O_2_/Ar plasma processing. Taking a look at the “off spot” survey spectra, one can distinguish an increase of the B1s/C1s line ratio together with the removal of adventitious N by the plasma treatment. Last but not least, the post-treatment “on spot” and “off spot” survey spectra display roughly the same intensities for the B1s, C1s, and O1s lines. A quantitative analysis of the B_4_C-related B1s and C1s XPS lines shown in Fig. S11 gives a B/C atomic ratio of 4.19, which is close to the expected stoichiometry of 4. Summarizing, this gives evidence for an efficient cleaning of the a-C spot, without a significant oxidation of the B_4_C layer that would be evident from an increase of the O1s line.

Regarding the foreign materials other than those to be expected from B_4_C, we note the occurrence of Au4f, N1s, and Fe2p lines (see Fig. S7). As the Au4f lines are present in the “off spot” survey spectrum, we attribute its occurrence due to an Au contamination during the B_4_C deposition process, while the N1s line is due to adventitious contaminations from exposure to the atmosphere being completely removed by the plasma process. In contrast to this, the Fe2p line - also being present in the “off spot” spectrum of the pristine material – is enhanced by the plasma process, thus raising the possibility of sputtering processes due to the heavy Ar species within the O_2_/Ar plasma.

#### X-Ray Reflectometry (XRR) analysis

**Figure 2:**
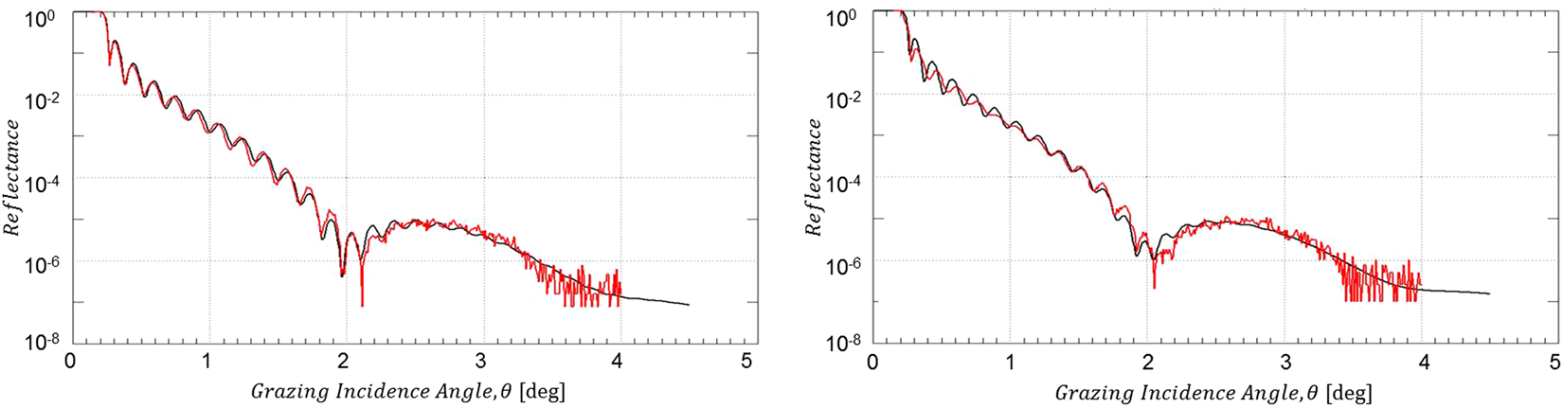
XRR data from B_4_C-coated test mirror right after O_2_/Ar plasma cleaning. Left hand side: Non a-C coated part; right hand side: Formerly a-C coated part (red solid lines: experimental XRR data; black solid lines: IMD simulation).

In order to probe the effect of the O_2_/Ar plasma treatment onto the integrity of the B_4_C coating and the roughness of the B_4_C/air interface, we have performed XRR measurements on both the B_4_C-coated test mirrors and the Si wafer test coupons. The results from both types of test specimen are depicted in Figs 2 and S14, respectively, and the parameters resulting from the IMD simulations of the experimental XRR data are given in Tables 3 and SII. All the XRR data simulation shown here include the Cr binding layer with about 2.2 nm thickness between the B_4_C coating and the Si substrate. XRR results for pristine as well as air-exposed Si test wafers can be found in section 3 of the Supplementary Information.

**Table 3:** Results from the IMD simulations of the XRR measurements as shown in Fig. 2 (bottom rows) as well as from reference measurements of non-processed samples (top rows).

Mirror test sample	B_4_C coating thickness [nm]	B_4_C/air interface rms roughness [nm]	B_4_C rms surface roughness [nm] (*)
After B_4_C deposition	**26.0**	**0.5–0.6**	**0.11**
After 7 months in air	26.9	~0.5	0.09
After O_2_/Ar plasma cleaning–non a-C coated part	25.1	~0.6	0.12
After O_2_/Ar plasma cleaning–a-C coated part	**25.9**	**~0.7**	**0.17**

(*)Surface roughness values from interference microscopy.

The O_2_/Ar plasma treatment of a B_4_C layer on a Si test wafer after an extended exposure to atmospheric air results in the removal of the physisorbed surface contaminations together with a reduction of the B_4_C layer thickness by 0.6 nm (i.e., 29.3 nm as compared to originally 29.9 nm). On the downside, the plasma treatment incurs an increase in rms surface roughness from 0.4 nm rms to 0.6 nm rms (see Table SII).

The XRR analysis from the B_4_C-coated mirror in the lower part of Table 3 basically conveys the same message: A reduction of the B_4_C layer thickness between 0.1 and 0.9 nm (for the formerly C-coated and non C-coated part, respectively) together with a slight increase of rms surface roughness between 0.1 and 0.2 nm.

The obvious differences in terms of absolute surface roughness numbers between the results obtained from XRR and interference microscopy (see Table 3) can be understood by the inherently different level of topographical sensitivity of these two techniques depending on the frequency range of the one-dimensional power spectral density function (PSD). The same argument applies when comparing, e.g., results from interference microscopy and AFM. Nevertheless, although being different in absolute size, both techniques should in principle observe the same trends.

**Figure 3:**
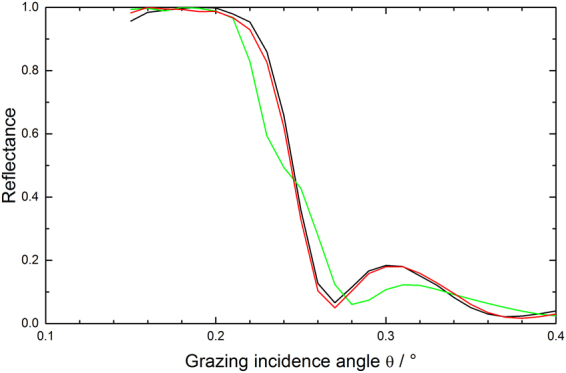
Comparison of XRR total reflection data at low grazing incidence angles for a pristine B_4_C-coated test mirror (black line) and after a-C coating plus subsequent O_2_/Ar plasma cleaning (green line: formerly a-C coated area; red line: non a-C coated area).

Additional information on the changes regarding especially the optical performance of the mirrors at Cu Kα wavelength for correspondingly larger penetration depths (i.e., as compared to XPS) can be obtained by evaluating the total reflection part of the XRR data at low grazing incidence angles. In that respect, Fig. 3 gives evidence for an incomplete carbon cleaning of the mirror surface due to the deviation of the spectrum from the formerly a-C coated mirror surface (green line) at about 0.25 degree grazing incidence as compared to the spectra for the pristine and non a-C coated samples (black and red lines, respectively). Also, the further discrepancies around 0.3 degree grazing incidence corroborate the changes in B_4_C layer thickness mentioned above. The results from the surface roughness measurements via interference microscopy give – although being quantitatively lower in absolute numbers than the XRR data – the same slight increase in terms of surface roughness by the plasma treatment (see Table 3). Especially the deviations of the reflectivity edge of the formerly a-C coated material as compared to the pristine reflectivity are far from acceptable in terms of the expected optical performance.

#### SEM results

**Figure 4:**
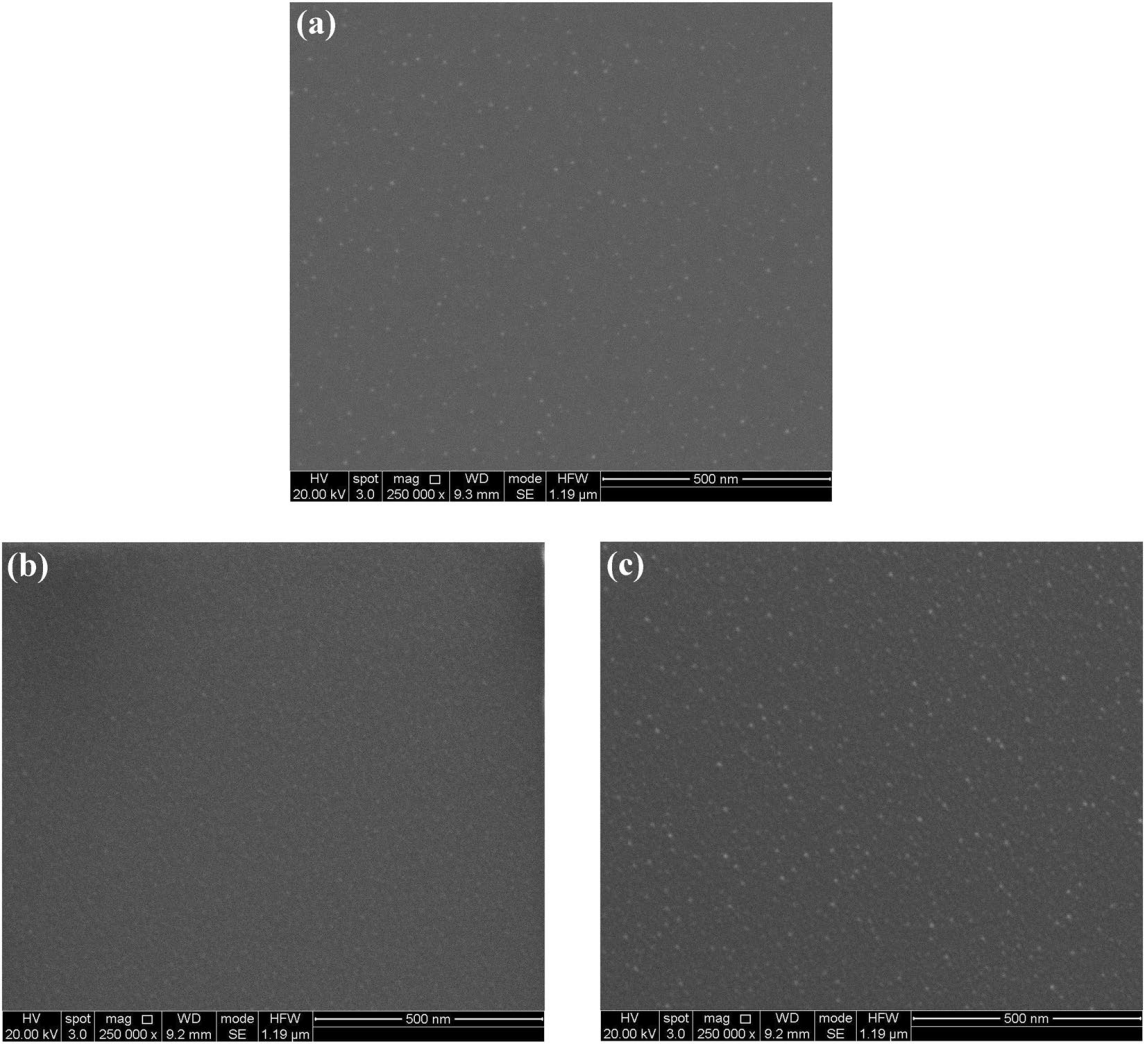
SEM images of B_4_C-coated test wafers taken at 20 kV electron acceleration voltage with a 250 k-fold magnification: (**a**) Pristine B_4_C-coated test wafer, (**b**) O_2_/Ar-plasma cleaned – formerly a-C coated part, and (**c**) O_2_/Ar-plasma cleaned – non a-C coated part.

The B_4_C surface morphology of the Si test wafers in the SEM images shown in Fig. 4 give a visual account for the slight increase in rms surface roughness mentioned above: Starting from the pristine test wafer in Fig. 4(a), one can distinguish small bright spots of about 10 nm lateral diameter that are distributed across the surface in an irregular manner. Similar “hillocks” – but of micrometric size - have been observed in other studies^13^ where they were attributed to crystalline B_4_C intergrowths in a B-doped pyrolytic carbon matrix formed during the post-growth heat treatment of B_4_C thin films as grown by CVD.

After a-C contamination and subsequent O_2_/Ar plasma treatment of the Si test wafer, there is a clear increase regarding the density of these hillocks in the SEM image in the non a-C coated part (see Fig. 4(c)) that obviously leads to the slight increase of the surface roughness from 0.5–0.6 nm rms to 0.6 nm rms as observed in the XRR data. Regarding Fig. 4(b), the visible residual carbon interface layer (see Fig. S3) apparently leads to lower density of these hillocks as compared to the formerly non a-C coated counterpart in Fig. 4(c), as they are apparently covered by the residual carbon interface. We attribute these hillocks to the formation of boron oxy-carbides that are actually more prominent in the case of B_4_C coatings treated with an Ar/H_2_ plasma that will be discussed in a later section.

Summarizing the results from the O_2_/Ar plasma cleaning of B_4_C-coated optical surfaces, this approach gives somewhat fair results in terms of preservation of the B_4_C layer thickness and morphology, but the carbon cleaning appears to be somewhat poor as evidenced by the total reflection XRR data as well as by the persistent non B_4_C-related contribution in the C1s XPS spectra (see Fig. 1).

### H_2_/Ar plasma cleaning

In this section, we follow the same approach as in the previous part, but this time using an H_2_/Ar feedstock gas plasma that has been successfully used for the cleaning of optical surfaces coated with non-noble metals such as, e.g., Rh or Ni that are inherently incompatible with an oxidizing plasma.

In Fig. S4, we show the a-C coated Si wafer and mirror test items before and after the H_2_/Ar plasma cleaning. According to the visual impression, the a-C residue appears to be less than in the case of the O_2_/Ar-plasma cleaned test objects (see Fig. S3). However, especially in the case of the test mirror the non a-C coated part appears to have lost some of the ocher color from the original B_4_C coating.

#### X-ray Photoelectron Spectroscopy (XPS) analysis

**Figure 5:**
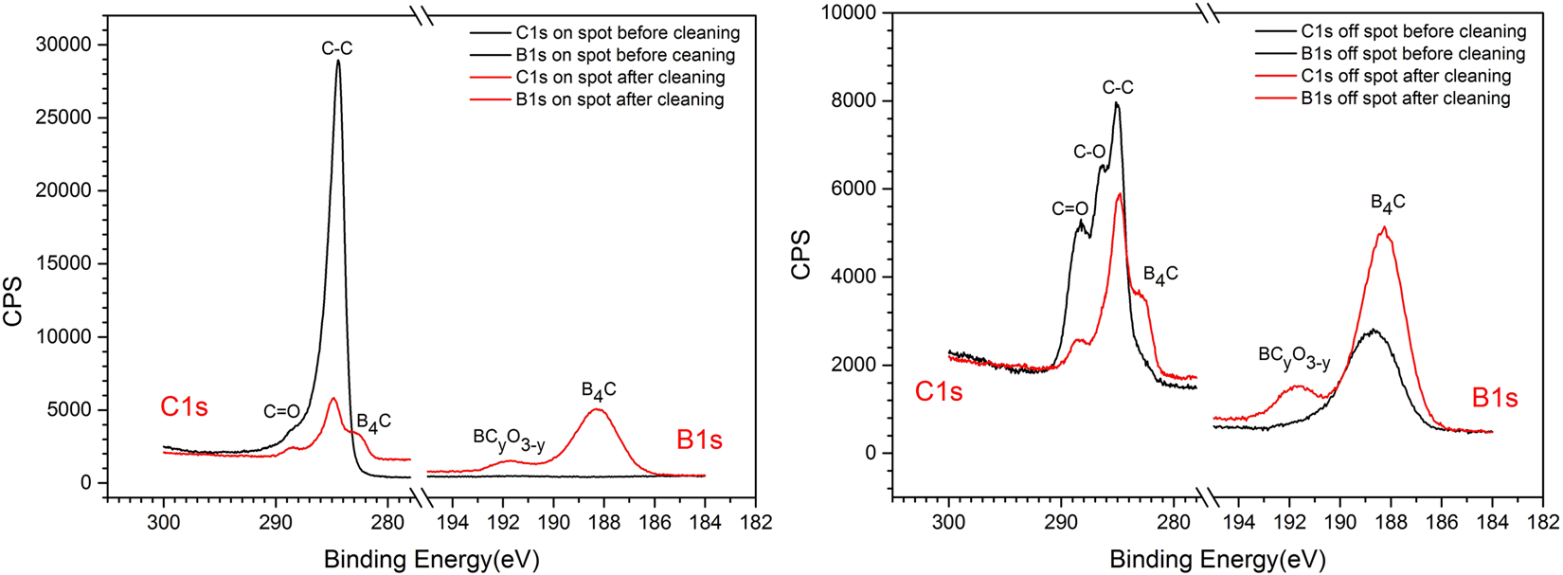
High-resolution C1s and B1s XPS spectra of the B_4_C-coated test wafer before (black solid lines) and after H_2_/Ar RF plasma cleaning (red solid lines). Left panel: XPS data taken on the amorphous carbon contamination spot. Right panel: XPS data taken off the amorphous carbon contamination spot on the bare B_4_C coating.

The high resolution XPS spectra in Fig. 5 yield the same results as their O_2_/Ar analogues in Fig. 1: An almost complete removal of the a-C contamination, including a persistent three peak structure in the C1s XPS range. However, we note that in this specific case a significant increase of the B1s peak at 191.6 eV B.E. occurs that we relate to boron oxy-carbides.

The XPS survey scans in Fig. S8 before/after H_2_/Ar plasma cleaning basically give the same results as in the case of the analogue spectra for the O_2_/Ar plasma treatment in Fig. S7 and also yield an efficient a-C removal from the sample surface. This resilience of the O1s line is surprising in a way as one would expect a decrease of the oxygen surface species due to the chemically reducing character of the H_2_/Ar plasma. However, a separate XPS depth profiling analysis (see Fig. S10) did reveal that the amorphous B_4_C layer contains a significant amount of oxygen, most probably as BC_2_O, BCO_2_, and B_2_O_3_ so that the simultaneously occurring removal of B_4_C surface layers (see below) leads to a persistent presence of oxygen species on the sample surface. As with the O_2_/Ar plasma appeared the Fe2p line, we attribute the latter to the plasma process, due to sputtering phenomena related with heavy Ar species within the H_2_/Ar plasma.

#### X-Ray Reflectometry (XRR) analysis

**Figure 6:**
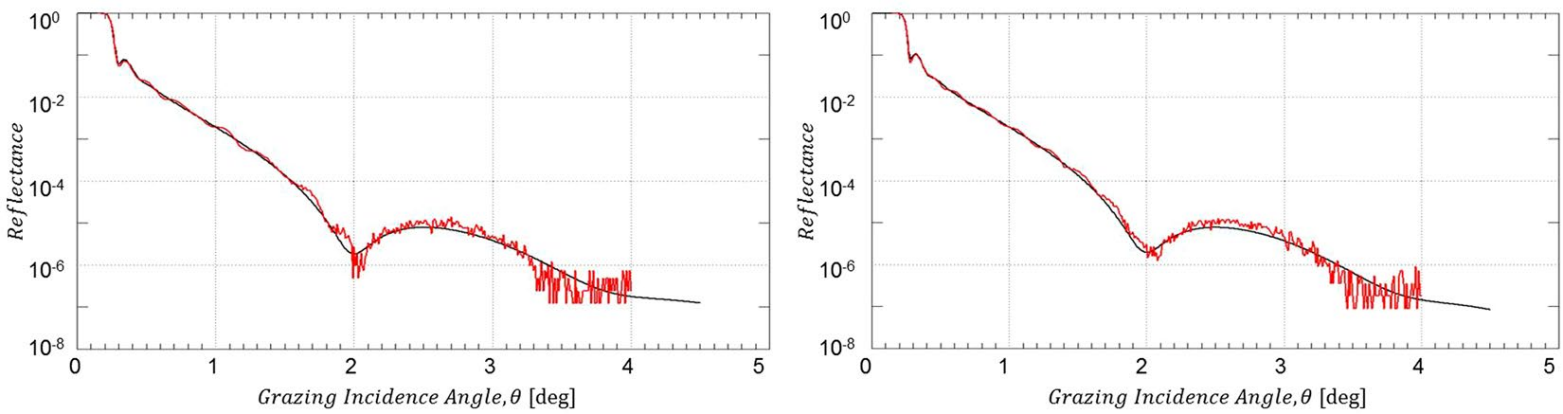
XRR data from B_4_C-coated test mirror right after H_2_/Ar plasma cleaning. Left hand side: Non a-C coated part; right hand side: Formerly a-C coated part (red solid lines: experimental XRR data; black solid lines: IMD simulation).

The significant drawbacks from the H_2_/Ar plasma cleaning become most apparent from the XRR measurements presented in Fig. 6. As already obvious from these data at first glance, there is an almost complete loss of Kiessig fringes throughout the complete angular range, which applies to both the non a-C coated and the formerly a-C coated part. Taking a closer look at the results from the IMD simulations in Table 4, one can conclude that there has been a substantial reduction of the B_4_C layer thickness by about 8 and 4 nm for the non a-C coated and the formerly a-C coated part, respectively, together with a large increase of the B_4_C/air interface roughness beyond 3 nm rms. These latter large numbers are probably partly due to some sample non-uniformities that may well contribute to the surface roughness figures. Nevertheless, the contribution from B_4_C surface roughness itself is still substantial.

**Table 4:** Results from the IMD simulations of the XRR measurements as shown in Fig. 6 (bottom rows) as well as from reference measurements from a non-processed sample (top row).

Mirror test sample	B_4_C coating thickness [nm]	B_4_C/air interface rms roughness [nm]	B_4_C rms surface roughness [nm] (*)
After B_4_C deposition	26.0	~0.5–0.6	0.12
After H_2_/Ar plasma cleaning–non a-C coated part	18.2	~3.4	0.15
After H_2_/Ar plasma cleaning–a-C coated part	21.7	~3.5	0.17

(*) Surface roughness values from interference microscopy.

The surface micro-roughness data from interference microscopy (see Table 4) give a quantitatively more realistic picture, but still indicating a significant increase by the plasma treatment.

**Figure 7:**
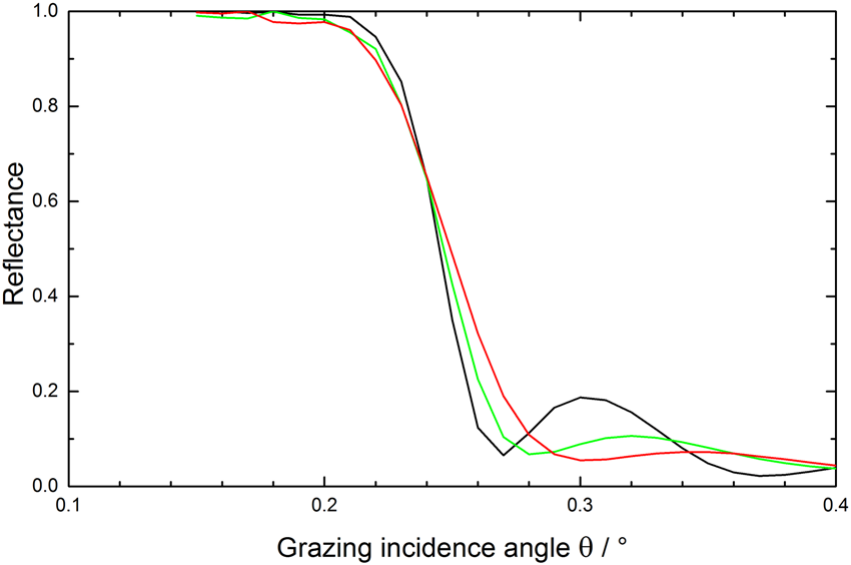
Comparison of XRR total reflection data at low grazing incidence angles for a pristine B_4_C-coated test mirror (black line) and after a-C coating plus subsequent H_2_/Ar plasma cleaning (green line: formerly a-C coated area; red line: non a-C coated area).

The total reflection data shown in Fig. 7 again indicate the structural changes for the B_4_C layer at grazing incidence angles beyond 0.26 degree, while from the chemical perspective the deviation in the XRR data at about 0.25 degree indicate a satisfactory cleaning, which does not come as a surprise taking into account the loss of B_4_C layer thickness mentioned above. Nevertheless, the clearly observable changes in the reflectivity edge for the plasma treated samples as compared to the pristine samples (i.e., distinct changes of the edge slope plus the occurrence of additional steps at about 0.18 degree grazing angle) indicate a severe and unacceptable change of the optical performance of the B_4_C coating due to, e.g., a chemical residue from the plasma cleaning, a change of the B_4_C density, and/or B_4_C surface roughness.

#### SEM results

**Figure 8:**
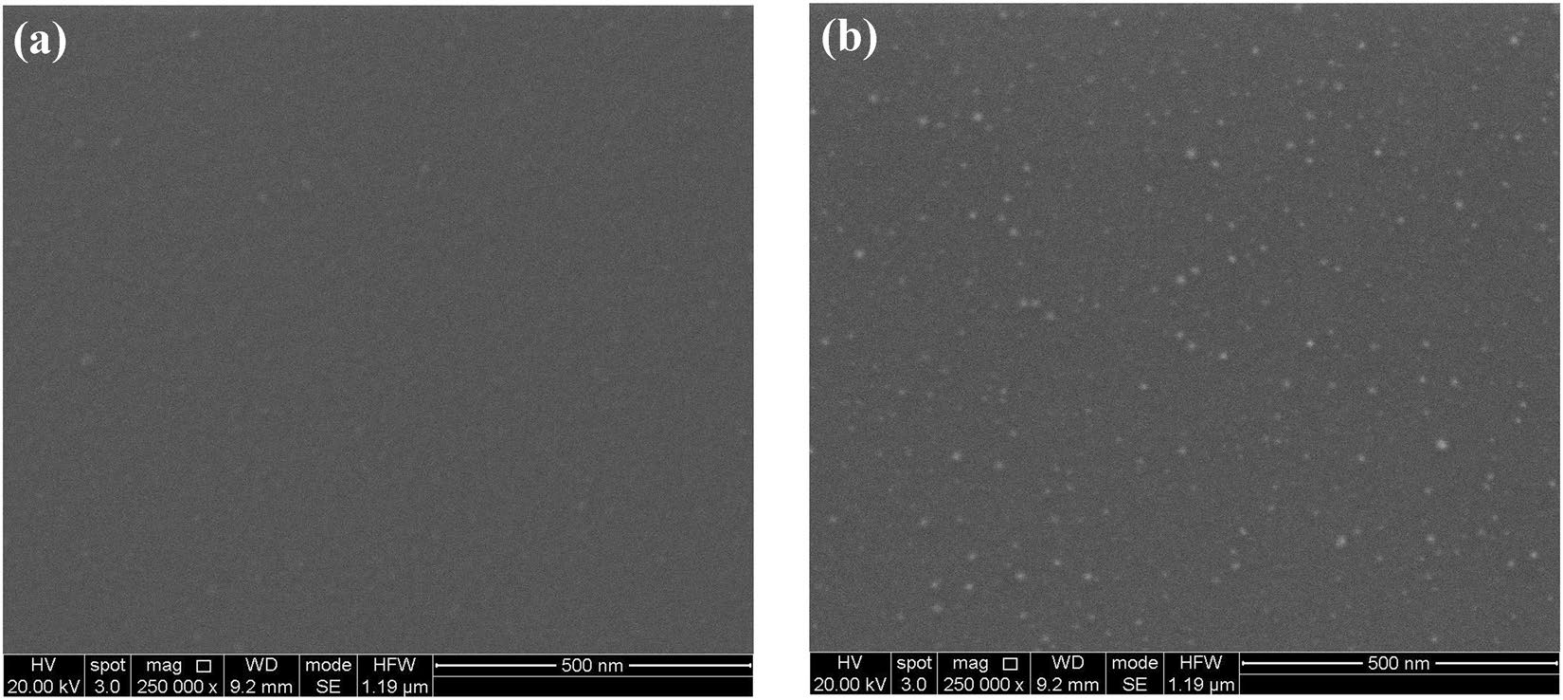
SEM images of B_4_C-coated test wafers taken at 20 kV electron acceleration voltage with a 250 k-fold magnification after cleaning with H_2_/Ar plasma: (**a**) Formerly a-C coated part, and (**b**) non a-C coated part.

The SEM results from a H_2_/Ar -cleaned B_4_C-coated test coupon are depicted in Fig. 8. Comparing these results with their analogues from the O_2_/Ar plasma cleaning in Fig. 4, the images taken in the formerly a-C coated part appear to be very similar (i.e., visually indicating a carbonaceous residue on the samples surface), whereas the non a-C coated part of the H_2_/Ar-cleaned B_4_C-coated test coupon exhibits fewer, but more distinct/larger hillocks that we attribute to the boron oxycarbides that appears more strongly in the B1s XPS line in Fig. 5 (“off spot” after cleaning). It is obviously somewhat surprising that these hillocks can still be observed after a reducing H_2_/Ar plasma process, but - as mentioned above - our XPS depth profile analysis in Fig. S10 shows that the B_4_C bulk material within the layers include quite some boron oxide phase that emerges to the surface during the sputtering by the Ar species within the plasma. Thus, we presume that there is a plasma-induced formation of boron oxy-carbide hillocks that are created by the interaction of the plasma with the B_2_O_3_ phases and B_4_C phases emerging at the sample surface.

Summarizing the results from the H_2_/Ar plasma cleaning of B_4_C-coated optical surfaces, this approach gives contrary results as compared to those from the O_2_/Ar plasma cleaning described in the previous section: Fair results in terms of carbon cleaning, but very bad results regarding the preservation of the B_4_C layer thickness and morphology as evidenced by the total refection XRR data.

### Pure O_2_ plasma cleaning

In this section, we describe the results from the low-pressure RF plasma cleaning using pure O_2_ feedstock gas. In Fig. S5, we show a B_4_C-coated Si test mirror during various stages of the experimental process. As can be seen from these photographs, the fully processed mirror compares visually quite well to the pristine mirror, which gives a first positive indication regarding the completeness of the cleaning process as well as the preservation of the B_4_C coating.

#### X-ray Photoelectron Spectroscopy (XPS) analysis

**Figure 9:**
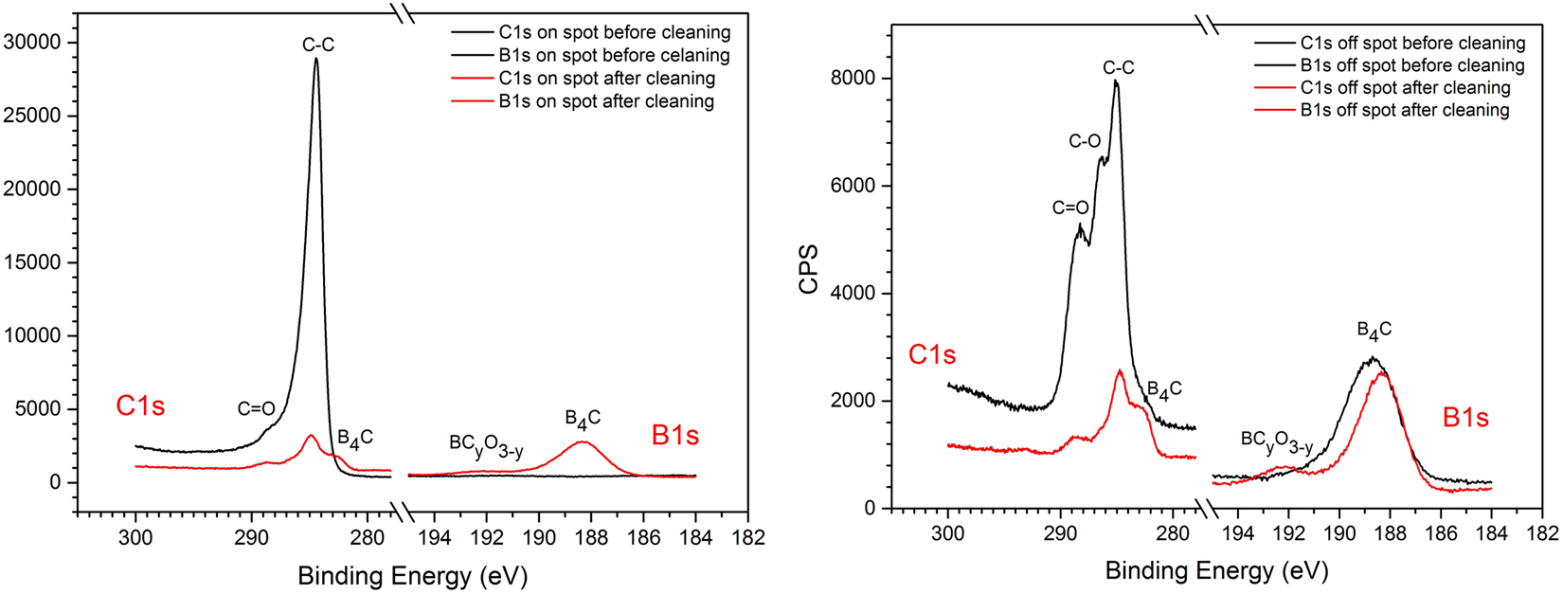
High-resolution C1s and B1s XPS spectra of the B_4_C-coated test wafer before (black solid lines) and after pure O_2_ RF plasma cleaning (red solid lines). Left panel: XPS data taken on the amorphous carbon contamination spot. Right panel: XPS data taken off the amorphous carbon contamination spot on the bare B_4_C coating.

In Fig. 9, we show the high resolution C1s and B1s XPS spectra taken off as well as on the a-C spot before and after the pure O_2_ plasma cleaning. As can be observed from the C1s spectra, there is a strong reduction of the C1s lines related to C=O, C-O, and C-C species for both the “off spot” and the “on spot” spectra due to the plasma cleaning process, whereas the B1s line to 188.3 eV B.E. related to B_4_C shows up after the plasma treatment. In the case of the B1s spectra, the “off spot” spectra exhibit a shift of the B1s line to lower B.E. (plus a decrease of the line width) and the appearance of a small additional peak at about 192.2 eV, that we attribute to oxidized boron and boron oxy-carbides, respectively.

Figure S9 shows the XPS survey spectra from B4C-coated Si test wafers with the XPS measurements being performed off as well as on the a-C spot before and after the pure O2 plasma cleaning. The “on spot” spectra give evidence for an efficient removal of the previously deposited a-C layer based on the significant reduction of the C1s line together with the appearance of the B1s line from the B4C layer. The same applies to the “off spot” spectra, although with an obviously less spectacular reduction of the C1s line (as we are dealing with “off spot” spectra).

Regarding foreign elements, we again note the occurrence of a small amount of Au4f lines that have been identified as intrinsic to the B_4_C coating as they are already present in the “off spot” spectrum of the sample before plasma treatment. It can also be noted that there is no Fe2p line visible in the survey spectra of the plasma-processed samples. This is in contrast to the previous section and is attributed to the fact that there has been no Ar gas added to the pure O_2_ feedstock gas mixture, which would otherwise lead to the aforementioned sputtering phenomena resulting in the deposition of a small amount of Fe.

#### X-Ray Reflectometry (XRR) analysis

**Figure 10:**
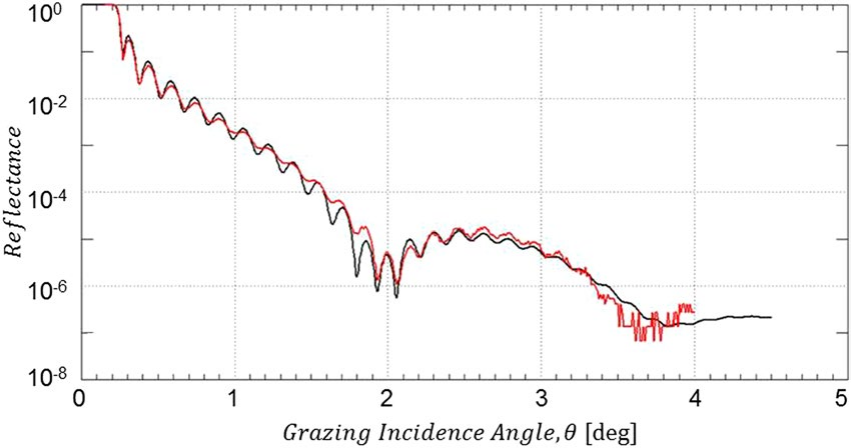
XRR data from B_4_C-coated test mirror after cleaning with pure O_2_ plasma cleaning. (red solid lines: experimental XRR data; black solid lines: IMD simulation).

The measured XRR data in Fig. 10 from the formerly fully a-C contaminated test mirror (see Fig. S5) together with the results from the IMD simulations given in Table 5 fully corroborate the results from the visual impression by the photographs in Fig. S5. As can be seen from the measured data, the Kiessig fringes throughout the full angular range are maintained and the parameters resulting from the pertinent IMD simulations yield a slight reduction of the B_4_C coating thickness (i.e., 25.5 nm as compared to 26.0 nm) together with an unaltered B_4_C/air interface roughness of 0.5 nm rms.

**Table 5:** Results from the IMD simulations of the XRR measurements as shown in Fig. 10 (bottom row) as well as from reference measurements of non-processed samples (top rows).

Mirror test sample	B_4_C coating thickness [nm]	B_4_C/air interface rms roughness [nm]	B_4_C rms surface roughness [nm] (*)
After B_4_C deposition	**26.0**	**~0.5–0.6**	**0.10**
After 7 months in air	27.0	~0.6	—
After pure O_2_ plasma cleaning–a-C coated part	**25.5**	**~0.5**	**0.26**

(*) Surface roughness values from interference microscopy.

On the other hand, the micro-roughness data from interference microscopy show a substantial increase from 0.1o to 0.26 nm rms, which is at variance with the results from XRR. We attribute these contrasting trends to the technical differences between these two surface characterization techniques that will be discussed in the context of the SEM images in this section.

**Figure 11:**
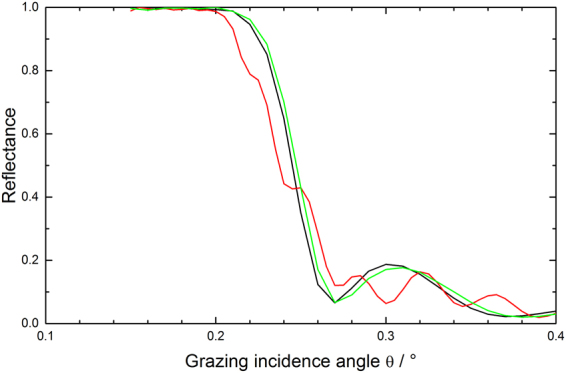
Comparison of XRR total reflection data at low grazing incidence angles for a pristine B_4_C-coated test mirror (black line), after a-C coating (red line), and after subsequent cleaning with pure O_2_ plasma (green line).

The XRR total reflection data from a B_4_C-coated test mirror in Fig. 11 give further support for a full recovery of the B_4_C surface at negligible losses regarding the integrity of the B_4_C layer. The spectrum for the a-C coated (i.e., non plasma-cleaned) mirror surface shows the expected chemical and structural deviations in the range from 0.2 to 0.26 degree and beyond 0.26 degree, respectively, due to the additional a-C top layer. On the other hand, the spectrum of the subsequently plasma cleaned sample nicely overlaps with the spectrum from the pristine untreated clean test mirror in terms of the shape and slope of the reflectivity edge, beside a small shift regarding the edge position to slightly higher angular values. This leads to the conclusion for a full chemical and structural recovery of the test mirror from the perspective of Cu Kα wavelengths and also applies to the optical performance of the fully processed mirror. Nevertheless, taking into account nowadays B_4_C optical coating manufacturing technologies a rms roughness figure of 0.26 nm as determined by interference microscopy (see Table 5) appears acceptable.

#### SEM results

**Figure 12:**
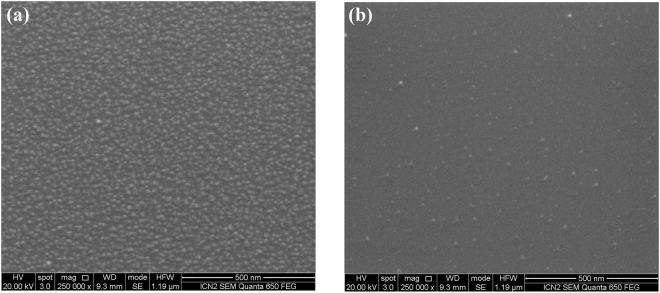
SEM images of B_4_C-coated test wafers taken at 20 kV electron acceleration voltage with a 250k-fold magnification after cleaning with pure O_2_ plasma: (**a**) Formerly a-C coated part, and (**b**) non a-C coated part.

In Fig. 12, we show the SEM images of a fully processed B_4_C-coated test coupon that has been a-C contaminated and subsequently cleaned with pure O_2_ plasma. What can be immediately concluded from the images is that whereas the appearance of the non a-C coated part in Fig. 12(b) is similar to that of the B_4_C reference sample in Fig. 4(a) as well as the corresponding images from the other plasma processed samples in Figs 4(c) and 8(b). In stark contrast to this, the image from the formerly a-C coated part in Fig. 12(a) exhibits a large density of small structures with diameters between 15 and 20 nm. As according the XPS results given in Fig. 9 the surface chemistry of the formerly a-C coated part is very similar to the ones from the O_2_/Ar and H_2_/Ar plasma treatment (see Figs. 1 and 5, respectively), we conclude that the surface modification in Fig. 12(a) is of a pure morphological nature, without apparently alternating the surface chemical characteristics that are basically given by the aforementioned plasma-induced conversion of the B_4_C and B_2_O_3_ bulk phases into boron oxy-carbides.

As mentioned above, the B_4_C/air interface roughness as measured with XRR remains unchanged (see Table 5), whereas interference microscopy gives an increase from 0.10 to 0.26 nm rms. Taking into account the rather enhanced density of hillocks in Fig. 12(a) - that equals an almost full surface coverage - together with the fact that XRR is measured at grazing incidence, it seems plausible that interference microscopy (close to normal incidence) gives the more reliable surface roughness information, consistent with the SEM results.

Summarizing the results from the pure O_2_ downstream plasma cleaning, we conclude that this approach is so far the only promising pathway within this study leading to an efficient cleaning together with the desirable preservation of the B_4_C surface morphology and chemistry, together with a conservation of the B_4_C bulk layer thickness. This also applies to the expected optical performance of the B_4_C coating as derived from the total reflection data. Nevertheless, the applied plasma cleaning time still has to be carefully taken into account in view of avoiding an over-cleaning and thus damaging of the B_4_C surface coating.

### Considerations on a-C plasma cleaning mechanisms and cleaning speed

In Table 6, we give a brief summary of the experimentally derived a-C cleaning rates that have been determined in the present study on a-C contaminated B_4_C-coated test objects presented here (extrapolated values) and on Au-coated QCM crystals that have been achieved in a previous study^7,8^.

**Table 6:** Experimentally derived a-C cleaning rates for different types of plasma feedstock gases for either B_4_C-coated Si test wafers and Au-coated QCM crystals.

Plasma type and cleaning mechanism	B_4_C coated test coupons (this study; extrapolated)	Au-coated QCM crystal (previous study*)
Pure O_2_ (chemical cleaning)	3.5 Å/min.	8.4 Å/min.
O_2_/Ar (mostly chemical cleaning)	4.6 Å/min.	11.6 Å/min.
H_2_/Ar (mostly kinetic cleaning)	2.3 Å/min.	1.7 Å/min.

All values were measured using 100 W plasma RF power at a plasma vacuum pressure of 0.005 mbar (*data taken from refs^7,8^).

As has been observed previously, there is a large difference by roughly a factor of 7 between the a-C cleaning rates that can be achieved using either O_2_/Ar or H_2_/Ar feedstock gases on metallic optical coatings and substrates. One the other hand, it is clear from these numbers that while going from Au to B_4_C coatings, the cleaning rates for the O_2_ and O_2_/Ar-based plasma are strongly reduced (roughly by a factor of 2 to 2.5), whereas the cleaning rates for the H_2_/Ar-based plasma is basically maintained.

We basically attribute these changes in a-C cleaning rates for the different substrate coatings – i.e., more specifically the lowering of cleaning rates for the O_2_-gas based plasma when going from Au to B_4_C coatings – to two different phenomena:

- The plasma-induced formation of a chemically resilient carbonaceous interface layer on the sample surface. This is corroborated by the observation of an ubiquitous non-removable and optically visible residue at the location of the former a-C contamination and its the resilience and, at the same time, the close similarity of the XPS spectra after the cleaning process.
- The fundamentally different processes that lead to the removal of the a-C contamination as a function of the chemical composition of the plasma feedstock gas. In the oxygen-rich/argon-poor O_2_ and O_2_/Ar plasma, we assign most of the cleaning to the chemical activity of the oxygen species within the plasma. On the other hand, in the case of the argon-rich H_2_/Ar plasma, a significant (or may be even dominant) part of the a-C removal is done via the kinetic effect from the Ar species in the plasma as well as possibly UV photochemical contributions from an Ar metastable state.

The above two phenomena apparently result in the reduction of carbon cleaning rates due to the chemical resilience of the plasma-induced carbonaceous BOC layer, especially in the case of the chemical cleaning using the oxygen-based plasma. In contrast to this, the cleaning speed by the argon-rich H_2_/Ar plasma is maintained as the kinetic cleaning is kept up also in the case of the carbon-contaminated B_4_C-coated optics, which is corroborated by our findings regarding the reduction of the B_4_C layer thickness due to the H_2_/Ar plasma as discussed above. The latter does obviously not necessarily exclude the reductive effect of the H^•^ radicals that could be observed for the cleaning a-C contaminated metal coatings and substrates, where a significant reduction of Ni_2_O_3_ and Rh_2_O_3_ surface layers to metallic Ni and Rh could be observed previously.

Our recent results regarding the analysis of the GV10x downstream plasma using Langmuir probe as well as mass spectroscopy diagnostics indicate that in contrast to prior expectations, almost no neutral oxygen radicals could be detected in the O_2_/Ar plasma. Instead, the by far dominating species consist of ionized oxygen molecules (O_2_^+^) and atomic oxygen cations (O^+^). Also, the kinetic energies of the argon, hydrogen, and oxygen species involved in the downstream plasma at 100 W RF power amount to roughly 40 eV. Thus, the future analysis of the plasma chemistry involved in the removal of the a-C deposits as well as in the plasma/surface interaction with the B_4_C layers will have to take into account these findings.

## Summary and Outlook

In this paper, we report on different approaches regarding the *chemically selective* low pressure RF downstream plasma cleaning of B_4_C-coated and amorphous carbon-contaminated optics by the variation of the plasma feedstock gas and thus the inherent plasma chemistry. We thereby also report on the first successful plasma cleaning of such optics, i.e., without incurring damage of the B_4_C optical coating and its surface resulting into a fully maintained optical performance.

In more detail, we have performed an extensive test series on various test objects using O_2_/Ar, H_2_/Ar and pure O_2_ gas mixtures as plasma feedstock gases in order to determine the optimum plasma chemistry for the cleaning of B_4_C optical surface coatings.

Based on our analysis regarding the remote ICP plasma used, we conclude that the O_2_/Ar plasma cleaning yields fair results in terms of the preservation of the B_4_C layer, while the removal of amorphous carbon contaminations can be considered as poor. On the other hand, the H_2_/Ar plasma treatment leads to a significant damage of the B_4_C coating – both regarding the layer thickness and surface roughness – while the removal of amorphous carbon contaminations seems to be fairly satisfactory. However, it has to be taken into account that the latter is apparently an obvious consequence from the former. Last but not least, the plasma cleaning using pure O_2_ feedstock gas yields excellent results regarding both the a-C cleaning as well as the preservation of the B_4_C layer. We would like to emphasize at this point that other cleaning approaches may well lead to comparable results regarding B_4_C cleaning but, to the best of our knowledge, no pertinent studies can be found in the present literature on this specific topic.

From the above observations, we conclude that the Ar species within the plasma – especially in conjunction with the reduced a-C cleaning rates and thus enhanced cleaning times of an H_2_/Ar plasma – result into sputtering phenomena on the B_4_C layers that lead to the observed reduction in layer thickness and enhanced surface roughness. Thus, a pure O_2_ feedstock gas plasma (i.e., without admixture of heavy Ar gas atoms) offers a damage-free and fast cleaning, based on the chemical activity of the O species. First measurements using plasma mass spectroscopy indicate that the predominant oxygen species within an O_2_/Ar feedstock gas plasma consist of O_2_^+^ species.

Almost independent from the plasma used, we have observed the plasma-induced formation of a fair amount of boron oxy-carbides on the B_4_C surfaces that presumably appear as hillocks on these surfaces and thus contribute to the surface micro-roughness. This especially applies to the H_2_/Ar plasma-cleaned test samples. The formation of this chemically rather resilient carbonaceous layer could be observed both visually as well as based on XPS data and appears to contribute to the required extended cleaning times as compared to, e.g., the cleaning of metallic optical coatings. Everything considered, we conjecture that the formation of this surface boron oxy-carbide layer is the result from the surface interaction of the plasma with the B_2_O_3_ and B_4_C phases inherent to the bulk of the B_4_C coating, resulting into the formation of the said surface boron oxy-carbides.

The apparent sensitivity of the B_4_C layers with respect to a plasma-induced damage (i.e., as compared to apparently less sensitive metallic optical coatings) emphasizes the importance of an *in situ* monitoring of the cleaning process in view of avoiding a cleaning process beyond the time required for the full removal of the carbon contaminations. To this end, an optical *in situ* end point detection scheme is presently under development that allows monitoring the progress of the cleaning process in order to stop the treatment once a clean optical surface has been achieved.

## Electronic supplementary material

Supplementary Material
