# Supplementary material for: Characterization of Carbon-Contaminated B4C-Coated Optics after Chemically Selective Cleaning with Low-Pressure RF Plasma
Source: Sci Rep. 2018 Jan 22;8:1293. doi: 10.1038/s41598-018-19273-6 (PMC5778011; doi:10.1038/s41598-018-19273-6)
Supplement: Supplementary file 1 — Supplementary Material [file 41598_2018_19273_MOESM1_ESM.pdf]

<u>Supplementary Material for:</u>

Characterization of Carbon-Contaminated B_4C-Coated Optics after Chemically Selective Cleaning with Low-Pressure RF Plasma

H. Moreno Fernández[a*], D. Rogler[b], G. Sauthier[c], M. Thomasset[d], R. Dietsch[b], V. Carlino[e], E. Pellegrin[a]
[a]CELLS-ALBA, Carrer de la Llum 2-26, E-08290 Cerdanyola del Valles, Spain
[b]AXO DRESDEN GmbH, D-01237 Dresden, Germany
[c]ICN2, UAB Campus, E-08193 Bellaterra, Spain
[d]SOLEIL Synchrotron, L'Orme des Merisiers, F-91192 Gif-sur-Yvette, France
[e]ibss Group Inc., Burlingame, CA 94010, USA

1. Si wafer and mirror test objects

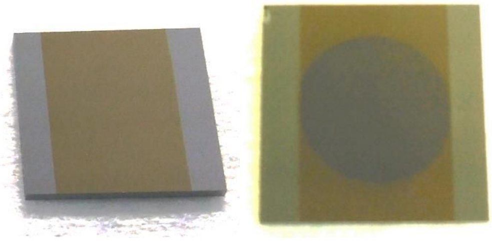

Fig. S1: Si(100) test wafers (10x10 mm^2 size) provided with a B_4C coating stripe (ocher color – see left hand side panel) plus a central amorphous carbon contamination spot (dark grey color – see right hand side panel) on top of the B_4C layer.

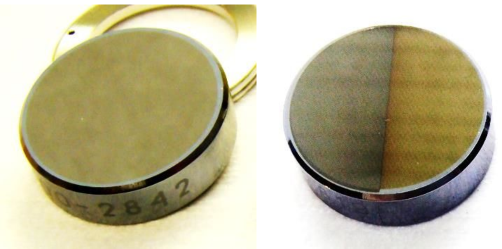

Fig. S2: Optically polished Si test mirrors with one inch diameter. The mirror on the left hand side shows a pristine B_4C coating, while the mirror on the right hand side exhibits one half of its surface (i.e., the darker part) coated with an additional amorphous carbon layer with 70 nm thickness.

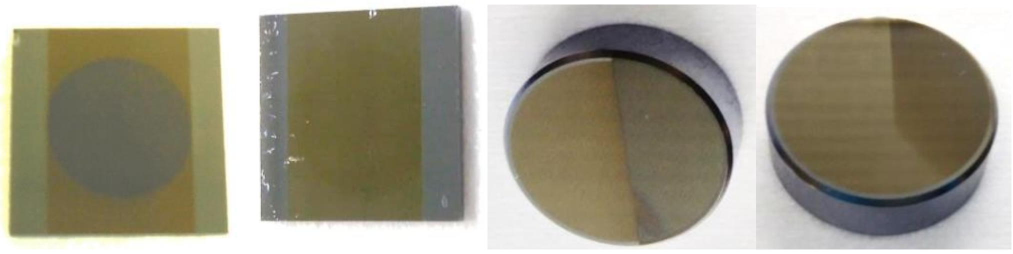

Fig. S3: B_4C-coated Si test wafers and test mirrors before (left) and after (right) O_2/Ar plasma cleaning.

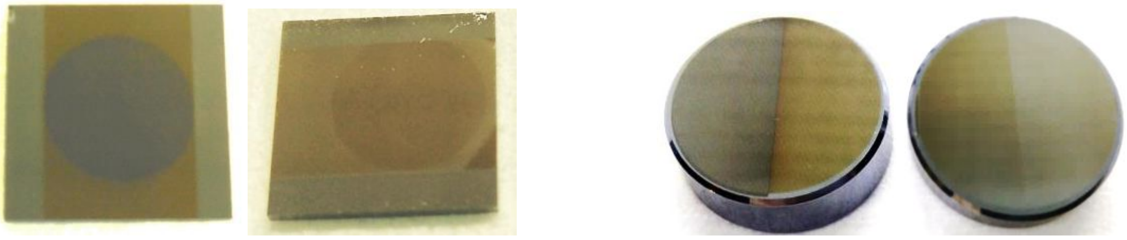

Fig. S4: a-C coated B_4C Si test wafers and test mirrors before (left) and after (right) H_2/Ar plasma cleaning.

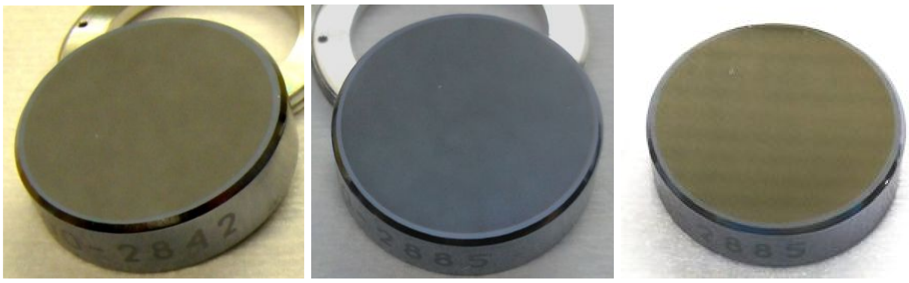

Fig. S5: B_4C-coated Si test mirrors during various stages of the process. Left: Pristine B_4C-coated Si mirror; Center: Mirror after a-C coating (full mirror surface coated); Right: Mirror after cleaning with pure O_2 plasma.

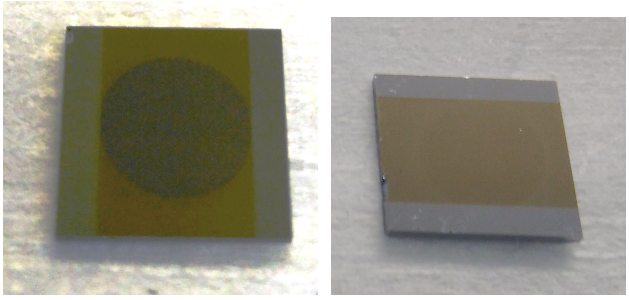

Fig. S6: a-C coated B_4C Si test wafers before (left) and after (right) pure O_2 plasma cleaning.

2. XPS analysis

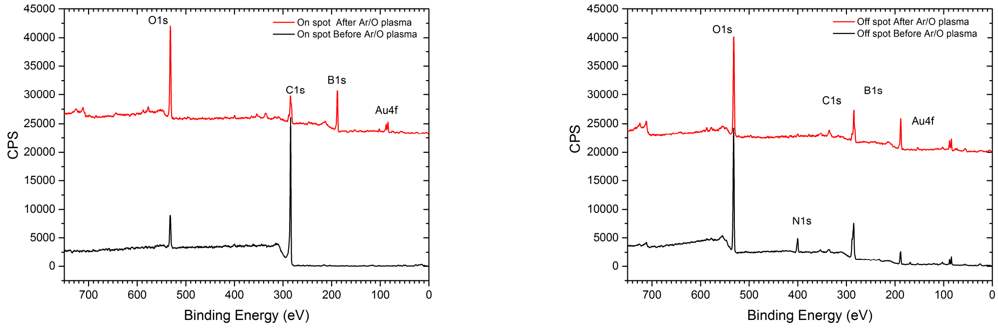

Fig. S7: XPS survey scans taken from B_4C-coated Si test wafers before (solid black lines) and after (solid red lines) O_2/Ar plasma cleaning. The left and right hand side diagram shows the XPS spectra taken at a sample location on and off the amorphous carbon spot, respectively.

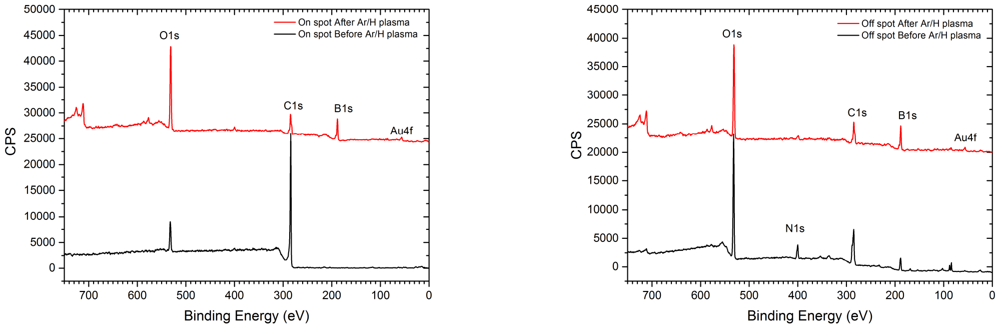

Fig. S8: XPS survey scans taken from B_4C-coated Si test wafers before (solid black lines) and after (solid red lines) H_2/Ar plasma cleaning. The left and right hand side diagram shows the XPS spectra taken at a sample location on and off the amorphous carbon spot, respectively.

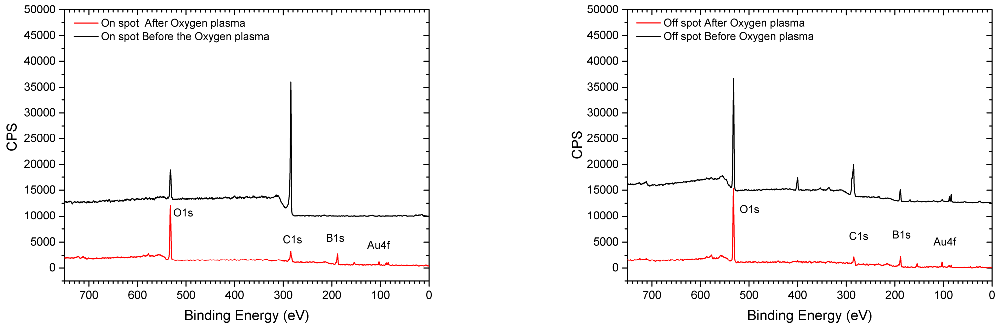

Fig. S9: XPS survey scans taken from B_4C-coated Si test wafers before (solid black lines) and after (solid red lines) pure O_2 plasma cleaning. The left and right hand side diagram shows the XPS spectra taken at a sample location on and off the amorphous carbon spot, respectively.

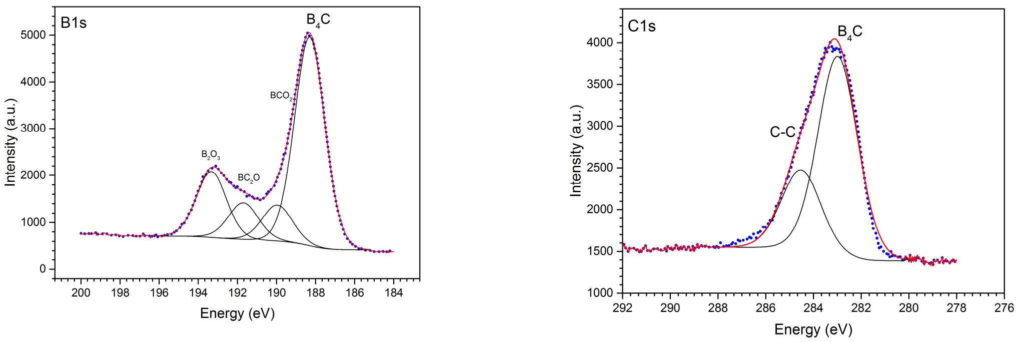

Fig. S10: XPS high resolution scans taken from a B_4C-coated Si test wafers after the 10 minutes of Ar sputtering (at 0.5 keV) performed in the XPS analysis chamber.

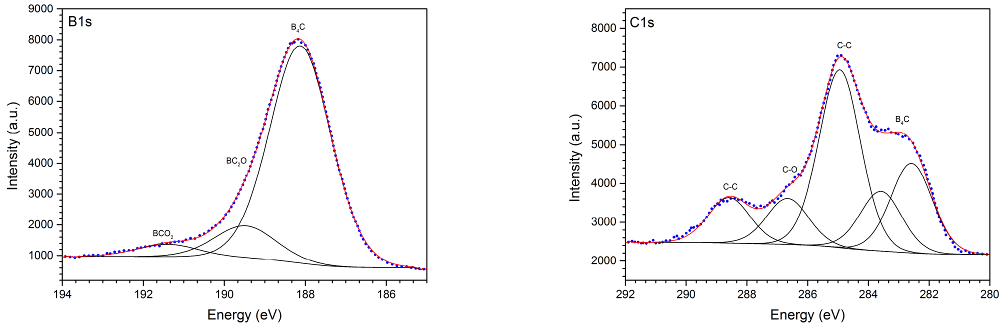

Fig. S11: XPS high resolution scans taken from a B_4C-coated Si test wafer after the O_2/Ar plasma cleaning. The left and right hand side diagram shows the B1s and the C1s XPS spectra taken at a sample spot corresponding to the location of the amorphous carbon spot.

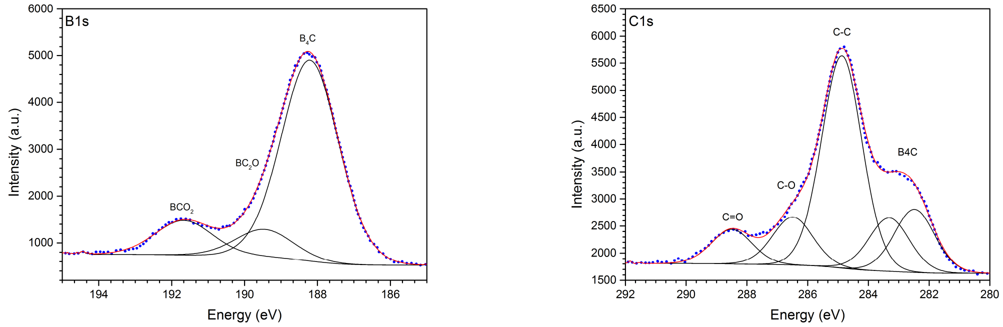

Fig. S12: XPS high resolution scans taken from a B_4C-coated Si test wafer after the H_2/Ar plasma cleaning. The left and right hand side diagram shows the B1s and the C1s XPS spectra taken at a sample spot corresponding to the location of the amorphous carbon spot.

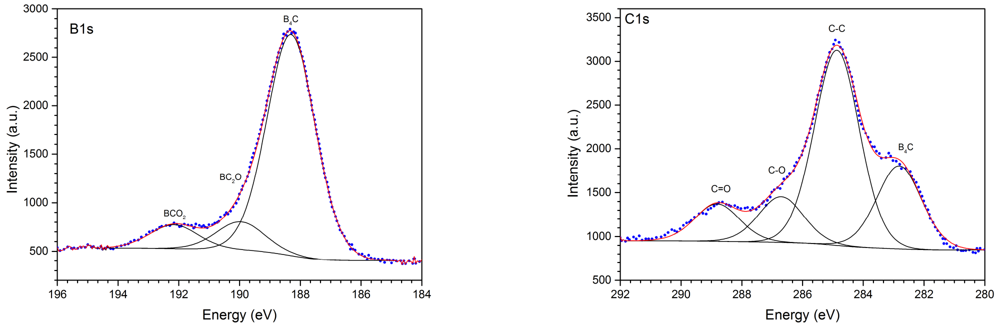

Fig. S13: XPS high resolution scans taken from a B_4C-coated Si test wafer after pure O_2 plasma cleaning. The left and right hand side diagram shows the B1s and the C1s XPS spectra taken at a sample spot corresponding to the location of the amorphous carbon spot.

**After 10 min Ar sputtering**		**After O_2/Ar plasma cleaning**		**After H_2/Ar plasma cleaning**		**After O_2 plasma cleaning**		**Peak assignment**
B1s FWHM:1.807	188.3	B1s FWHM 1.777	188.12	B1s FWHM:1.856	188.21	B1s FWHM:1.868	188.30	B-B and B-C in B_4C
	189.95		189.49		189.46		189.97	B in BC_2O
	191.69		191.40		191.66		192.16	B in BCO_2
	193.33		--		--		--	B in B_2O_3
C1s FWHM 1.954	282.98	C1s FWHM 1.1616	282.58 & 283.58	C1s FWHM:1.566	282.48 & 283.31	C1s FWHM:1.740	282.82	C in B_4C
	284.52		284.94		284.87		284.87	C-C
	--		286.66		286.49		286.71	C-O (e.g., in BOC)
	--		288.58		288.50		288.81	C=O

Table SI: Deconvolution of the fitted B1s and C1s high resolution XPS spectra in Figs. S10 to S13 for different surface treatments. All peak energy and full width at half maximum (FWHM) values are given in eV ("BOC" refers to boron oxy-carbides).

3. XRR analysis on B_4C-coated Si test wafers

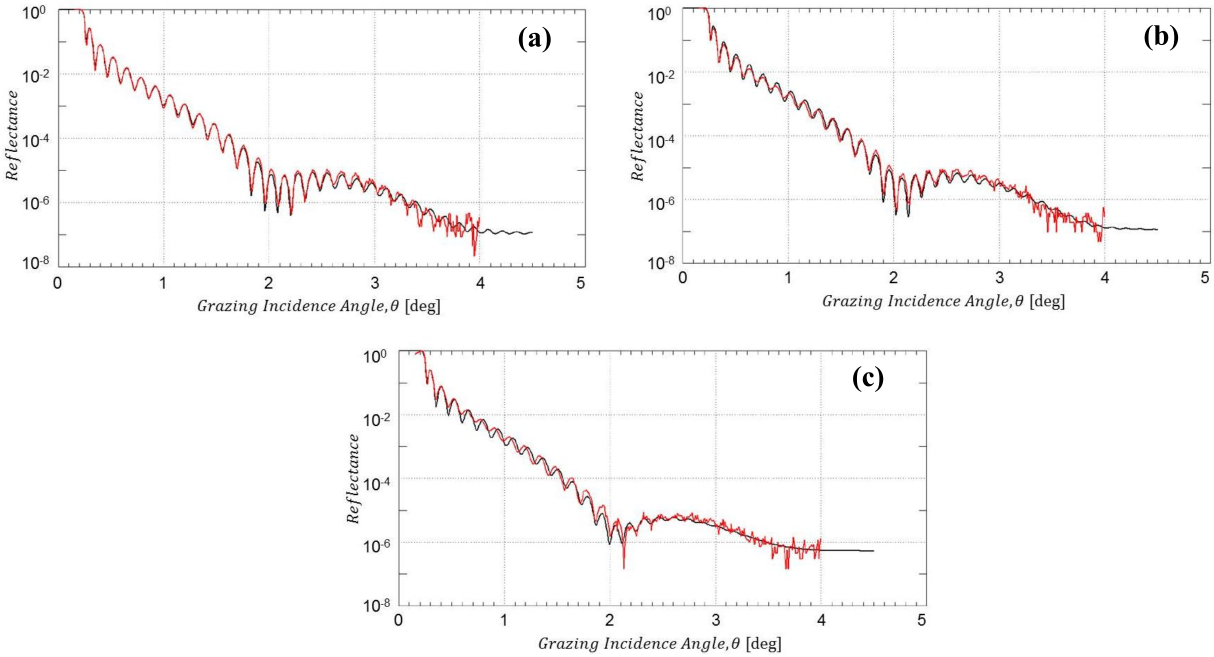

Fig. S14: XRR data from (a) pristine B_4C-coated Si test wafer right after B_4C deposition, (b) after 7 months of storage in air, and (c) after O_2/Ar plasma cleaning (red solid lines: experimental XRR data; black solid lines: IMD simulation)

Si wafer test sample	B_4C coating thickness [nm]	B_4C/air interface rms roughness [nm]
After B_4C deposition	**29.9**	**~ 0.4**
After 7 months in air	31.0	~ 0.5
After O_2/Ar plasma cleaning	**29.3**	**~ 0.6**

Table SII: Results from the IMD simulations of the XRR measurements as shown in Fig. S14.

When comparing the XRR results from the pristine Si test wafer (i.e., measured right after the B_4C deposition) with those from the Si wafer stored in air for 7 months, there is an apparent increase in B_4C layer thickness (i.e., 31.0 nm as compared to 29.9 nm). This is ascribed to the oxidation of the B_4C surface due to the exposure to atmospheric air, leading to the formation of BC_2O and BCO_2 together with the adsorption of adventitious carbon from atmospheric gases. Due to the limited chemical selectivity/sensitivity of the XRR analysis, we are left with the resulting apparent increase of the B_4C layer by roughly 1.1 nm.
